# Handedness Side and Magnetization Transfer Ratio in the Primary Sensorimotor Cortex Central Sulcus

**DOI:** 10.1155/2019/5610849

**Published:** 2019-08-05

**Authors:** Mario Mascalchi, Stefano Ciulli, Andrea Bianchi, Chiara Marzi, Stefano Orsolini, Gioele Gavazzi, Marco Aiello, Emanuele Nicolai, Andrea Soricelli, Marco Giannelli, Stefano Diciotti

**Affiliations:** ^1^“Mario Serio” Department of Experimental and Clinical Biomedical Sciences, University of Florence, Florence, Italy; ^2^Department of Electrical, Electronic, and Information Engineering “Guglielmo Marconi,” University of Bologna, Bologna, Italy; ^3^IRCSS Fondazione SDN, Naples, Italy; ^4^University of Naples Parthenope, Naples, Italy; ^5^Unit of Medical Physics, Pisa University Hospital “Azienda Ospedaliero-Universitaria Pisana,” Pisa, Italy

## Abstract

Left-handers show lower asymmetry in manual ability when compared to right-handers. Unlike right-handers, left-handers do not show larger deactivation of the ipsilateral primary sensorimotor (SM1) cortex on functional magnetic resonance imaging when moving their dominant than their nondominant hand. However, it should be noted that morphometric MRI studies have reported no differences between right-handers and left-handers in volume, thickness, or surface area of the SM1 cortex. In this regard, magnetization transfer (MT) imaging is a technique with the potential to provide information on microstructural organization and macromolecular content of tissue. In particular, MT ratio index of the brain gray matter is assumed to reflect the variable content of afferent or efferent myelinated fibers, with higher MT ratio values being associated with increased fibers number or degree of myelination. The aim of this study was hence to assess, for the first time, through quantitative MT ratio measurements, potential differences in microstructural organization/characteristics of SM1 cortex between left- and right-handers, which could underlay handedness side. Nine left-handed and 9 right-handed healthy subjects, as determined by the Edinburgh handedness inventory, were examined with T_1_-weighted and MT-weighted imaging on a 3 T scanner. The hands of subjects were kept still during all acquisitions. Using FreeSurfer suite and the automatic anatomical labeling parcellations defined by the Destrieux atlas, we measured MT ratio, as well as cortical thickness, in three regions of interests corresponding to the precentral gyrus, the central sulcus, and the postcentral gyrus in the bilateral SM1 cortex. No significant difference between left- and right-handers was revealed in the thickness of the three partitions of the SM1 cortex. However, left-handers showed a significantly (p = 0.007) lower MT ratio (31.92%  ± 0.96%) in the right SM1 central sulcus (i.e., the hand representation area for left-handers) as compared to right-handers (33.28%  ± 0.94%). The results of this preliminary study indicate that quantitative MT imaging, unlike conventional morphometric MRI measurements, can be a useful tool to reveal, in SM1 cortex, potential microstructural correlates of handedness side.

## 1. Introduction

Lateralization, i.e., structural or functional difference between the left and the right cerebral hemisphere of the brain, is a fundamental principle of brain organization in vertebrate and perhaps also in invertebrate animals [1, 2]. Hypothetical advantages of lateralization include sparing of neuronal activity duplication in the two hemispheres and skip of slow callosal transfer of signals between the two cerebral hemispheres [2]. Handedness is one of the most investigated manifestations of lateralization in the human brain and is related to the lateralized organization of cognitive systems [2]. Roughly ten percent of people are mixed- or left-handers and they generally show reductions or reversals of some cerebral functional asymmetries compared to right-handers [3].

Magnetic resonance imaging (MRI) has been used to explore possible brain functional and structural correlates of handedness side [3–8]. Functional MRI (fMRI) studies based on the blood oxygenation level dependent (BOLD) contrast have shown that the significantly larger deactivation of the ipsilateral primary sensorimotor (SM1) cortex when moving their dominant than their nondominant hand, typically observed in right-handers, is not present in left-handers [5, 8]. However, previous structural MRI studies have reported no differences between right-handers and left-handers in volume, thickness, or surface area of SM1 cortex, especially in the central sulcus [3, 6, 7, 9, 10] where hand motor function is localized according to the homunculus [3, 7]. Only one study has reported an increased cortical thickness in the right superior temporal gyrus in the left-handed subjects as compared to right-handed subjects [7].

Magnetization transfer (MT) is a physical process which can be exploited to obtain a specific contrast in MRI. In particular, quantitative MT imaging can be a useful tool to infer information on microstructural organization and macromolecular content of brain tissue [11]. MT is based on the exchange of magnetization between “free” protons and “bound” protons [12]. Free protons are the protons from water in the liquid phase (i.e., liquid pool), which are prevalent in the body and provide signal in conventional MRI. Bound protons represent protons embedded in proteins and macromolecules in relatively solid phases (i.e., macromolecular pool), which have a fairly broad magnetic resonance frequency spectrum. Given their low T_2_ (i.e., less than 1 ms), bound protons cannot be directly imaged in conventional MRI [13]. However, when a strong radiofrequency pulse with an offset from the magnetic resonance frequency of the liquid pool saturates selectively the magnetization of the macromolecular pool (MT weighting), there is a magnetization transfer from the liquid pool to the macromolecular pool [14]. This effect, which can depend on various biophysical factors, allows obtaining MT-weighted images (with reduced signal in regions characterized by a higher bound protons fraction) and its entity can be expressed by the MT ratio index (estimated by acquiring an image with and without MT weighting) [15]. In particular, MT ratio is higher in the white matter (WM) than in the gray matter (GM) of the normal brain. This is attributed to the higher protein content of myelin sheaths variably covering the neuronal axons of nerve fibers [12, 16–18]. While the higher MT effect explains the predominant applications of MT imaging to evaluate normal development and diseases of the WM [11], several studies have employed MT contrast for studying also normal [17, 19, 20] and disease states [11] of cortical and subcortical GM.

So far, no study has performed quantitative MT imaging measurements in SM1 cortex of left-handed and right-handed subjects. This preliminary study aimed hence at assessing, through MT ratio index as well as cortical thickness measurements in various SM1 cortex regions, potential differences in organization/characteristics of SM1 cortex tissue between left- and right-handers, which could underlay handedness side and are unlikely to be revealed by conventional morphometric MRI measurements.

## 2. Materials and Methods

### 2.1. Subjects

This study was performed according to the Declaration of Helsinki principles and approved by the Local Ethics Committee. Only healthy subjects who provided consent after explanation of the aims of the investigation were enrolled in the study. The study was based on 18 subjects (12 women and 6 men, mean age 25.3 ± 7.7 years) without familiar or personal history of neurologic or psychiatric disorders, selected from a wider population of 60 consecutive eligible healthy subjects undergoing MRI as controls for other clinical studies. In particular, they were selected on the basis of the assessment through the Edinburgh handedness inventory [21, 22]. This evaluates, for a subject, the dominance of right or left hand in 10 everyday activities. These activities include writing, drawing, throwing object, cutting with scissors, usage of toothbrush, cutting with knife (without fork) while eating, eating with spoon, usage of broom (considering the top hand) for cleaning the floor, striking match, and opening box. All the eligible subjects were interviewed by a single operator who registered the subject's answers assigning a score of null (no preference) or 1 to 2 (when the preference is so strong that you would never try to use the other hand) to each hand for each task. The lateralization index was then calculated [22]. In order to avoid a potential confounding effect due to mixed handedness side, we excluded 12 subjects exhibiting lateralization index comprised in the range [−70,70], which is a range wider than that (i.e., [−40,40]) indicated by Oldfield et al. [22] as the cut-off for ambidextrous subjects, and recruited a total of 9 left-handed (6 women and 3 men, mean age 24.9 ± 8.4 years, and mean lateralization index -86.6 ± 12.2) and 9 right-handed subjects (6 women and 3 men, mean age 25.7 ± 7.4 years, and mean lateralization index 92.2 ± 9.7). The latter were properly selected from the wider subpopulation of right-handers in order to match the left-handers for sex (p = 1.00 at the chi square test) and age (p = 0.84 at the two-tailed t-test).

### 2.2. MRI Acquisition Protocol

The subjects underwent MRI examination on a 3 T scanner system (Achieva, Release 2.6.3.7, Philips, Best, The Netherlands) with 33 mT/m maximum gradient strength and an 8-channel head coil.

After the scout image, an anatomical high resolution sagittal 3D T1-weighted turbo field echo (TFE) gradient-echo sequence [repetition time (TR) = 7.7 ms, echo time (TE) = 3.8 ms, flip angle = 8°, inversion time (TI) = 0 ms, field of view (FOV) = 256 mm × 256 mm, matrix size = 256 × 256, 160 continuous slices, slice thickness = 1 mm, number of excitations (NEX) = 1, and acceleration factor (SENSE) = 2] was acquired.

In order to obtain MT ratio maps, an axial 3D fast field echo (FFE) gradient-echo sequence [TR = 70 ms, TE = 2.7 ms, flip angle = 18°, FOV = 256 mm × 256 mm, matrix size = 172 × 172, 100 contiguous slices, slice thickness = 1.5 mm, NEX = 1, acceleration factor (SENSE) = 2], without (M0) and with (Ms) Gaussian sinc-shaped off-resonance pulse (bandwidth = 342 Hz, offset frequency = 1100 Hz, and duration = 17.5 ms), was acquired.

The acquisition time of the entire protocol was approximately 30 minutes. The hands of subjects enrolled in the study were kept still during all acquisitions.

### 2.3. MT Ratio Maps Computation

For each subject, Ms images were coregistered to M0 images by using a 6 degrees of freedom affine transformation implemented in FLIRT (FMRIB's Linear Image Registration Tool [23], as part of FSL [24]). Then, MT ratio maps were calculated voxel-wise as follows: [(M0–Ms)/M0]. A brain mask was also obtained by applying the FSL tool BET (Brain Extraction Tool [24, 25]) to M0 images.

### 2.4. Cortical Segmentation and Parcellation

A completely automated cortical segmentation and parcellation of each subject were performed by means of T_1_-weighted images and FreeSurfer image analysis suite v. 5.3 (http://surfer.nmr.mgh.harvard.edu/) [26]. Briefly, this includes removal of nonbrain tissue using a hybrid watershed/surface deformation procedure, automated Talairach transformation, segmentation of the subcortical WM and deep GM volumetric structures, intensity normalization, tessellation of the GM/WM boundary, automated topology correction [27], and surface deformation, following intensity gradients to optimally place the GM/WM and GM/cerebrospinal fluid borders at the location where the greatest shift in intensity defines the transition to the other tissue class.

**Figure 1:**
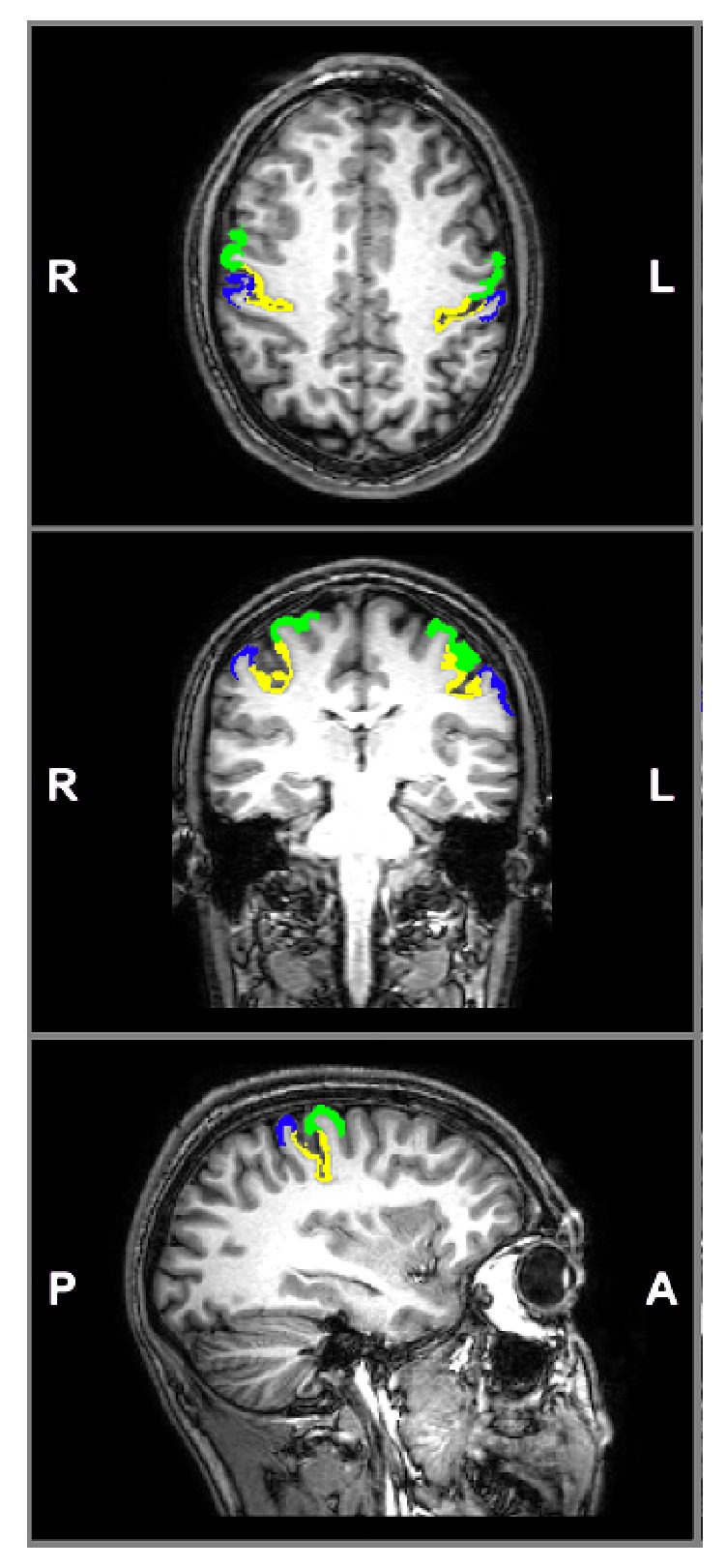
Axial, coronal and sagittal views of a sample FreeSurfer segmentation showing the precentral gyrus (green), central sulcus (yellow), and postcentral gyrus (blue) left and right ROIs defined by the Destrieux parcellation, overlaid to the T_1_-weighted image of the same subject. Sagittal view shows only the right ROIs.

We considered the FreeSurfer automatic anatomical labeling parcellations defined by the Destrieux atlas which includes, within each (left and right) SM1 cortical area, three regions of interests (ROIs) corresponding to the precentral gyrus, the central sulcus, and the postcentral gyrus [28] (Figure 1). Average thickness values within each single ROI were calculated by using FreeSurfer.

### 2.5. MT Ratio of SM1 Cortex ROIs

Using FLIRT, M0 images were affinely coregistered by means of 12 degrees of freedom transformation to the T_1_-weighted image in the native space of the subject. The ROI segmentations obtained from each subject were converted from the FreeSurfer space back to the native anatomical space. Then, the average of MT ratio values within each single ROI in left and right SM1 cortex was obtained.

### 2.6. Data Analysis and Repeatability Assessment

Any differences between left- and right-handers in terms of average MT ratio, as well as average thickness, in the 6 left and right ROIs covering the SM1 cortex, were assessed by means of the Mann-Whitney U test with Holm-Bonferroni correction for multiple comparisons (significance threshold of 0.05), in order to control the family wise error rate.

One volunteer right-handed subject (female, 36 years) was acquired 3 times by using the MT imaging protocol. For each ROI in the SM1 cortex, the coefficient of variation for repeated measurements of MT ratio (CV_MTR_) was calculated as the standard deviation value divided by mean value across repeated measurements.

## 3. Results

**Figure 2:**
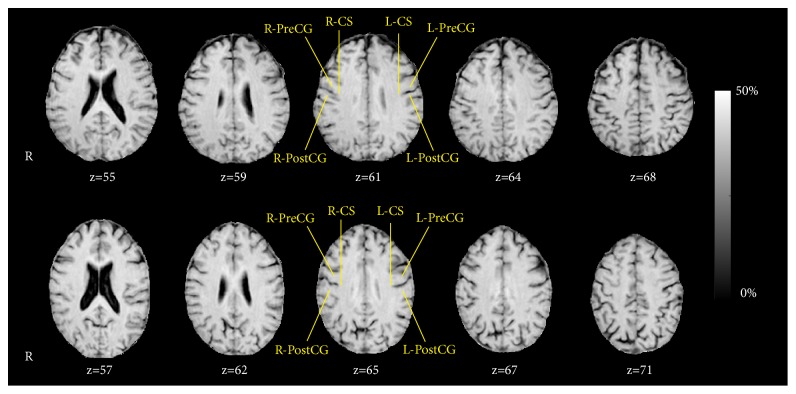
Example of MT ratio (%) maps in the native space (slice levels z, in voxels, encompassing the SM1 areas) for one representative left- (top row) and right-handed (bottom row) subject. The SM1 cortex areas of right precentral gyrus (R-PreCG), right central sulcus (R-CS), right postcentral gyrus (R-PostCG), left precentral gyrus (L-PreCG), left central sulcus (L-CS), and left postcentral gyrus (L-PostCG) are indicated.

Figure 2 shows representative MT ratio maps for a left-handed subject and a right-handed subject. Thickness and MT ratio results in the left- and right-handers are reported in Table 1. For all ROIs in SM1 cortex, thickness was not significantly different between left- and right-handers. On the contrary, left-handers showed a significantly lower MT ratio in the right SM1 central sulcus as compared to right-handers. No other significant differences were found.

As for the repeatability of MT ratio measurements, CV_MTR_ values (left/right) for central sulcus, precentral gyrus, and postcentral gyrus were 0.8%/1.9%, 3.6%/9.3%, and 5.2%/10.7%, respectively.

## 4. Discussion

Previous fMRI studies have extensively investigated the functional activities of the SM1 cortex during hand or finger movements. In unilateral hand movements, there is the evidence of a joint contralateral SM1 cortex activation and ipsilateral SM1 cortex deactivation [29]. FMRI and electrophysiological studies have consistently shown an asymmetry of the inhibition exerted by the contralateral toward the ipsilateral motor cortex during hand movements in right-handers (i.e., the left motor cortex has relatively greater effect on the right motor cortex) [30–32]. Also, in an fMRI study during execution of finger tapping alternatively with the right and left hand, right-handers exhibited significantly larger deactivation of the ipsilateral SM1 cortex when moving their dominant hand than their nondominant hand [5]. On the other hand, left-handers showed comparable ipsilateral SM1 cortex deactivation during either hand movement, consistent with left-handers' good performances with the right hand [8]. Overall, the results of these studies suggest that ipsilateral deactivation is a marker of hand lateralization [5, 8].

To the best of our knowledge, this is the first study assessing whether quantitative MT imaging can provide further insights into difference in microstructural organization of SM1 cortex between left- and right-handed subjects. We note that hands movement during data acquisition might affect MT ratio values and comparison between left- and right-handers. In this regard, some studies have shown a possible interaction between magnetization transfer and neuronal activation, neuronal depolarization and hypercapnia/hypoxia [33–35], albeit the specific mechanism is not yet well established. An interaction between magnetization transfer and blood oxygenation level dependent (BOLD) contrast has been found in functional magnetic resonance imaging studies [36]. In particular, Zhang et al. [37] have found variation of MT ratio values in neuronal activation area during a hand motor task. Given that our study is not a functional magnetic resonance imaging study and considering the above interaction between magnetization transfer and various factors such as hands movement and blood oxygenation, in order to introduce no potential bias in MT ratio measurements, the hands of subjects were kept still during all acquisitions.

We found that MT ratio was significantly lower in the right SM1 central sulcus in left-handers as compared to right-handers. It should be noted that, according to the Destrieux atlas, the central sulcus ROI encompasses Brodmann's areas 4 (precentral gyrus) and 1, 2, and 3 (postcentral gyrus), substantially matching the hand functional area as revealed by fMRI [6, 38]. On the contrary, we observed no significant difference in cortical thickness of SM1 ROIs between left- and right-handers, in line with previous conventional morphometric MRI studies of thickness as well as of volume and surface area [3, 6, 7, 9, 10]. Overall, these results indicate that MT ratio, given its dependence on various biophysical factors at microscopic level [11–14], can outperform conventional morphometric MRI measurements such as cortical thickness for revealing potential subtle differences in characteristics/organization of SM1 cortex tissue associated with handedness side.

**Figure 3:**
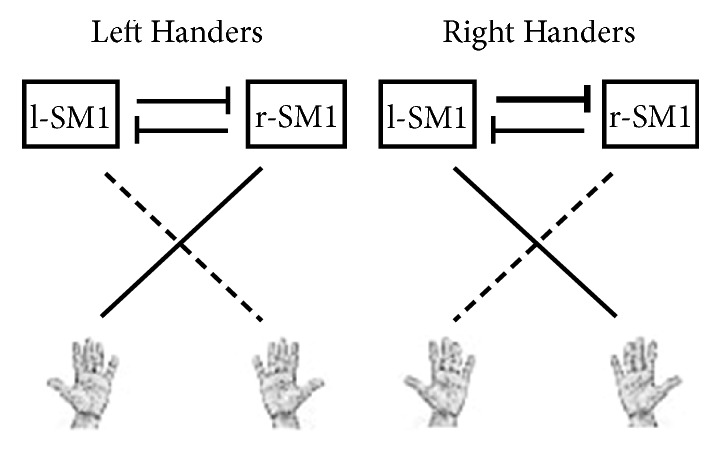
Scheme for possible asymmetric transcallosal inhibition underlying different MT ratio values in SM1 central sulcus between left- and right-handers. In left-handers, the right SM1 central sulcus may receive a lower number of transcallosal inhibitory fibers from the left SM1 (thin horizontal line) as compared to the right SM1 central sulcus in right handers (bold horizontal line). This would result in a lower cortical density of inhibitory fibers in left handers as compared to right-handers, yielding a lower MT ratio (thin vertical line) in the right SM1 central sulcus when compared to right handers (bold vertical line). Hand dominance is represented by the bold slanted line.

MT ratio of the brain GM is assumed to reflect the variable content of afferent or efferent myelinated fibers, with higher values being associated with increased number of fibers or degree of myelination [11, 39]. Therefore, our results in terms of MT ratio can represent a microstructural correlate of handedness side, which may suggest a lower sprouting of transcallosal myelinated fibers (Figure 3) in SM1 cortex central sulcus of left-handers as compared to right-handers. However, this speculative hypothesis on characteristics of transcallosal myelinated fibers would need further tailored studies to be demonstrated; in this regard, Gaussian and non-Gaussian diffusion MRI studies [40–43] could be useful to better infer microstructural properties of brain tissue associated with handedness side. Nonetheless, this hypothesis is potentially compatible with findings reported by previous fMRI studies showing the lack in left-handers of a significantly larger deactivation of the ipsilateral SM1 cortex when moving their dominant than their nondominant hand, a phenomenon that instead is typically observed in right-handers [5, 8]. Indeed, unilateral hand movements are associated with contralateral cerebral activation and ipsilateral cerebral deactivation, which has been supposed to result from an inhibitory effect exerted by transcallosal fibers [29, 44–46], albeit this has been refuted by one study performed on 3 right-handed patients with agenesis of the corpus callosum [47].

While an accurate assessment of repeatability of MT ratio measurements would require repeated acquisitions of a number of left- and right-handed subjects [48, 49], the CV_MTR_ values obtained for a single right-handed subject can still be of practical interest and provide an indicative estimation of repeatability. In particular, CV_MTR_ values of central sulcus ROIs were less than 2% (left 0.8%, right 1.9%) and rather lower than CV_MTR_ values of precentral (left 3.6%, right 9.3%) and postcentral (left 5.2%, right 10.7%) gyrus ROIs. This, along with the relatively low number of subjects enrolled in this study, could explain why we found significant difference in MT ratio between left- and right-handers only in the right central sulcus region of SM1 cortex.

We recognize as a potential limitation of this preliminary study the relatively low number of actually enrolled subjects in MT ratio analysis. Indeed, in order to avoid potential confounding effect due to mixed handedness side, we conservatively excluded 12 subjects exhibiting low/medium lateralization index.

## 5. Conclusions

This preliminary study shows that the MT ratio in the right SM1 cortex central sulcus is lower in left-handers than in right-handers, indicating that quantitative MT imaging can be a useful tool to reveal in SM1 cortex potential microstructural correlates of handedness side.

## Figures and Tables

**Table 1:** Cortical thickness and MT ratio values are expressed in terms of mean ± standard deviation, evaluated within the ROIs representing the primary sensory motor cortex parcellation, according to the Destrieux atlas. For each ROI, the p-value of the Mann-Whitney U test, performed to assess any significant difference in cortical thickness and MT ratio between left- and right-handers, is reported.

Region of interest	Thickness			MT ratio		
	(mm)			(%)		
	Left-handers	Right-handers	p-value	Left-handers	Right-handers	p-value
Left precentral gyrus	2.76 ± 0.18	2.74 ± 0.16	0.79	27.71 ± 4.65	28.87 ± 2.05	0.54
Left central sulcus	1.86 ± 0.17	1.89 ± 0.08	0.72	33.27 ± 4.65	33.74 ± 1.26	0.16
Left postcentral gyrus	2.07 ± 0.26	1.97 ± 0.11	0.38	28.23 ± 5.53	28.45 ± 2.41	0.72
Right precentral gyrus	2.80 ± 0.15	2.82 ± 0.17	0.72	27.92 ± 2.96	30.09 ± 2.01	0.13
Right central sulcus	1.87 ± 0.16	1.86 ± 0.07	1.00	31.92 ± 0.96	33.28 ± 0.94	0.007*∗*
Right postcentral gyrus	2.07 ± 0.31	1.95 ± 0.13	0.38	26.65 ± 3.34	28.50 ± 2.41	0.38

*∗* Significant after Holm-Bonferroni correction for multiple comparisons using a threshold value of 0.05.

## Data Availability

The data used to support the findings of this study are available from the corresponding author upon request.
