# Corrigendum to “Handedness Side and Magnetization Transfer Ratio in the Primary Sensorimotor Cortex Central Sulcus”

**DOI:** 10.1155/2020/3050735

**Published:** 2020-06-27

**Authors:** Mario Mascalchi, Stefano Ciulli, Andrea Bianchi, Chiara Marzi, Stefano Orsolini, Gioele Gavazzi, Marco Aiello, Emanuele Nicolai, Andrea Soricelli, Marco Giannelli, Stefano Diciotti

**Affiliations:** ^1^“Mario Serio” Department of Experimental and Clinical Biomedical Sciences, University of Florence, Florence, Italy; ^2^Department of Electrical, Electronic and Information Engineering “Guglielmo Marconi”, University of Bologna, Bologna, Italy; ^3^IRCCS SDN, Naples, Italy; ^4^University of Naples Parthenope, Naples, Italy; ^5^Unit of Medical Physics, Pisa University Hospital “Azienda Ospedaliero-Universitaria Pisana”, Pisa, Italy

In the article titled “Handedness side and magnetization transfer ratio in the primary sensorimotor cortex central sulcus” [1], affiliation 3 was incorrect. The correct affiliation is “IRCCS SDN, Naples, Italy,” and it is corrected above.
